# MRI Features of Intravenous Leiomyomatosis

**DOI:** 10.5334/jbsr.4201

**Published:** 2026-02-04

**Authors:** Beatriz Freixo, Delfim Doutel, Teresa Margarida Cunha

**Affiliations:** 1 Department of Radiology, Portuguese Institute of Oncology Francisco Gentil, Oporto, Portugal; 2 Department of Pathology, Portuguese Institute of Oncology Francisco Gentil, Lisbon, Portugal; 3 Department of Radiology, Portuguese Institute of Oncology Francisco Gentil, Lisbon, Portugal

**Keywords:** intravenous leiomyomatosis, uterine tumor, MRI, intravascular growth, differential diagnosis

## Abstract

Intravenous leiomyomatosis is a rare benign uterine smooth muscle tumor with intravascular growth through pelvic veins or lymphatics. A case is reported of a 42-year-old woman with intermittent spotting and progressive abdominal enlargement. Pelvic magnetic resonance imaging showed a heterogeneous myometrial mass with serpiginous extensions along iliac veins. The lesion had high signal on high *b*-value diffusion-weighted images and high apparent diffusion coefficient values, indicating the absence of diffusion restriction. After surgery, histopathology confirmed the diagnosis of intravenous leiomyomatosis.

*Teaching point:* Recognition of serpiginous intravascular extensions and the absence of diffusion restriction on magnetic resonance imaging should raise suspicion for intravenous leiomyomatosis.

## Introduction

Intravascular leiomyomatosis (IVL) is a rare benign tumor with an unusual intravascular growth pattern that may mimic malignant behavior. It was first described by Birsh-Hirschfeld in 1896 and later defined by Norris and Parmley in 1975 [1]. Fewer than 400 cases have been documented [2].

IVL represents benign smooth muscle proliferation within venous or lymphatic channels, arising either from direct extension of a uterine leiomyoma or from vascular intimal smooth muscle proliferation. Myometrial and broad-ligament venous channels are usually first involved. From there, extensions may reach in 10–40% of patients the right atrium or pulmonary artery via systemic venous circulation [3].

Contributing factors include prior uterine surgery, venous stasis, and increased estrogen exposure [2]. Symptoms include pelvic discomfort or fullness. Extra-pelvic involvement may cause lower-limb edema, dyspnea, or cardiopulmonary compromise, though patients may remain asymptomatic even in advanced stages [3].

## Case Report

A 42-year-old woman presented with intermittent spotting and progressive abdominal enlargement. Past surgical history included left salpingectomy for ectopic pregnancy and left ovarian cystectomy by laparotomy. Physical examination revealed a distended abdomen and a markedly enlarged, mobile uterus.

**Figure 1:**
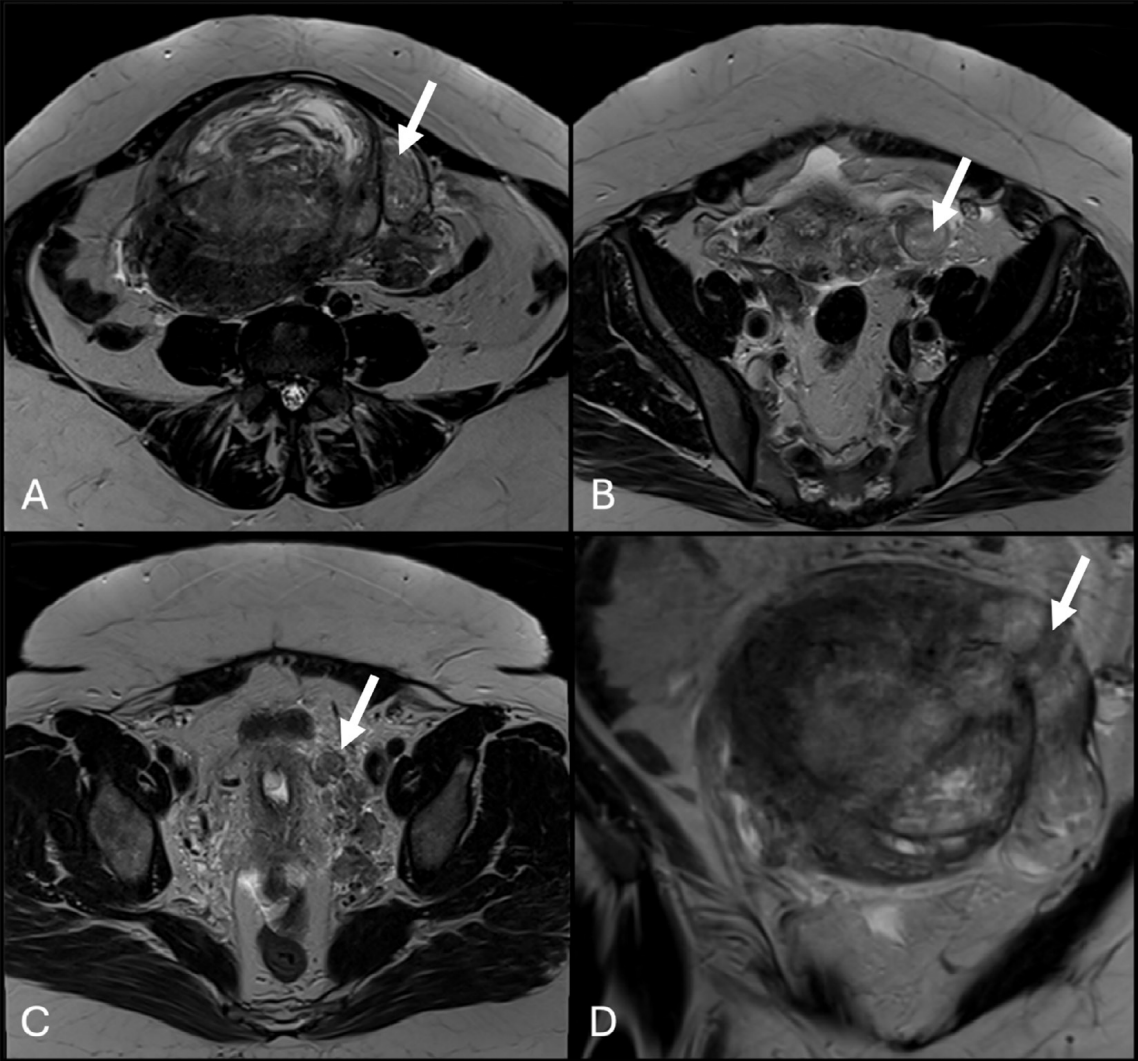
Axial **(A–C)** and coronal **(D)** T2-weighted images showing a large heterogeneous myometrial mass with lobulated contours and serpiginous extensions (arrows). Axial images **(A–C)** represent different levels of the tumor from superior to inferior.

**Figure 2:**
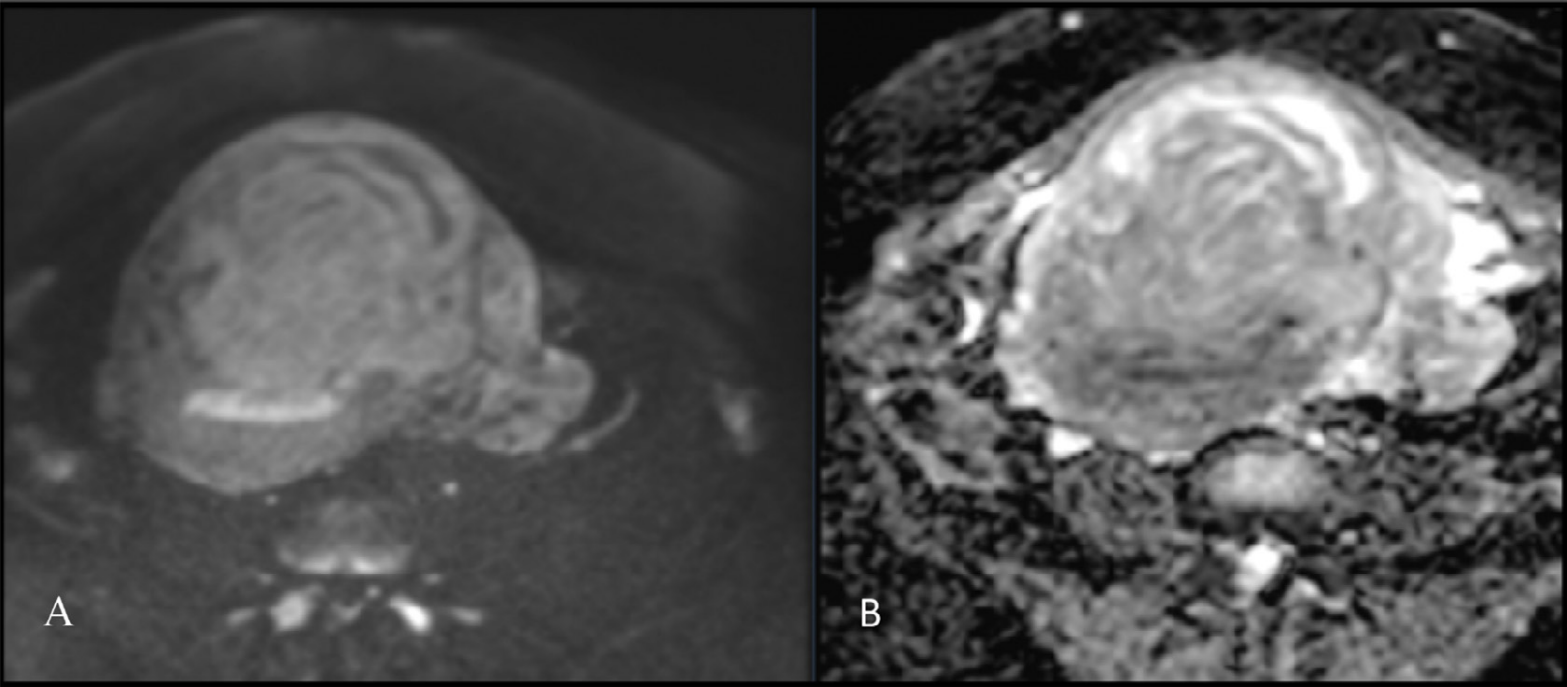
Axial DWI (*b* = 1000 s/mm²) **(A)** and corresponding ADC map **(B)** demonstrating the myometrial mass with high signal intensity on DWI and correspondingly high ADC values, indicating no diffusion restriction.

Pelvic magnetic resonance imaging (MRI) showed a heterogeneous myometrial mass with lobulated contours and serpiginous extensions following parametrial vessels (Figure 1). Diffusion-weighted imaging (DWI) showed high signal intensity on high *b*-value images with high apparent diffusion coefficient (ADC) values, consistent with absence of restricted diffusion (Figure 2).

Thoracic and abdominal computed tomography excluded lymphadenopathies and distant metastases. Serum tumor markers were within normal limits, and cytoreductive surgery was performed.

**Figure 3:**
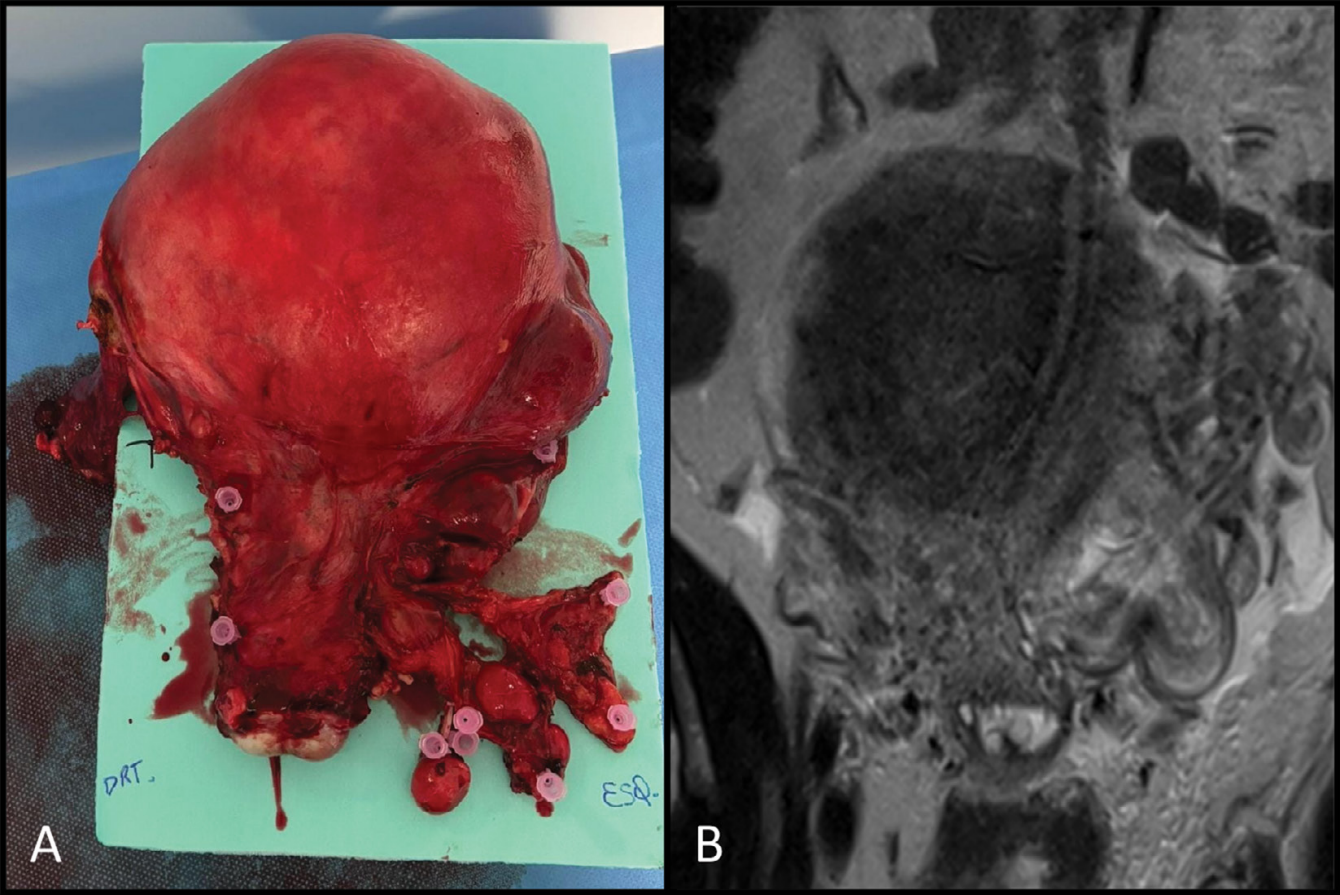
Surgical specimen **(A)** and coronal T2-weighted image **(B)** showing a uterus distorted by multiple nodules, extending into the left parametrium.

**Figure 4:**
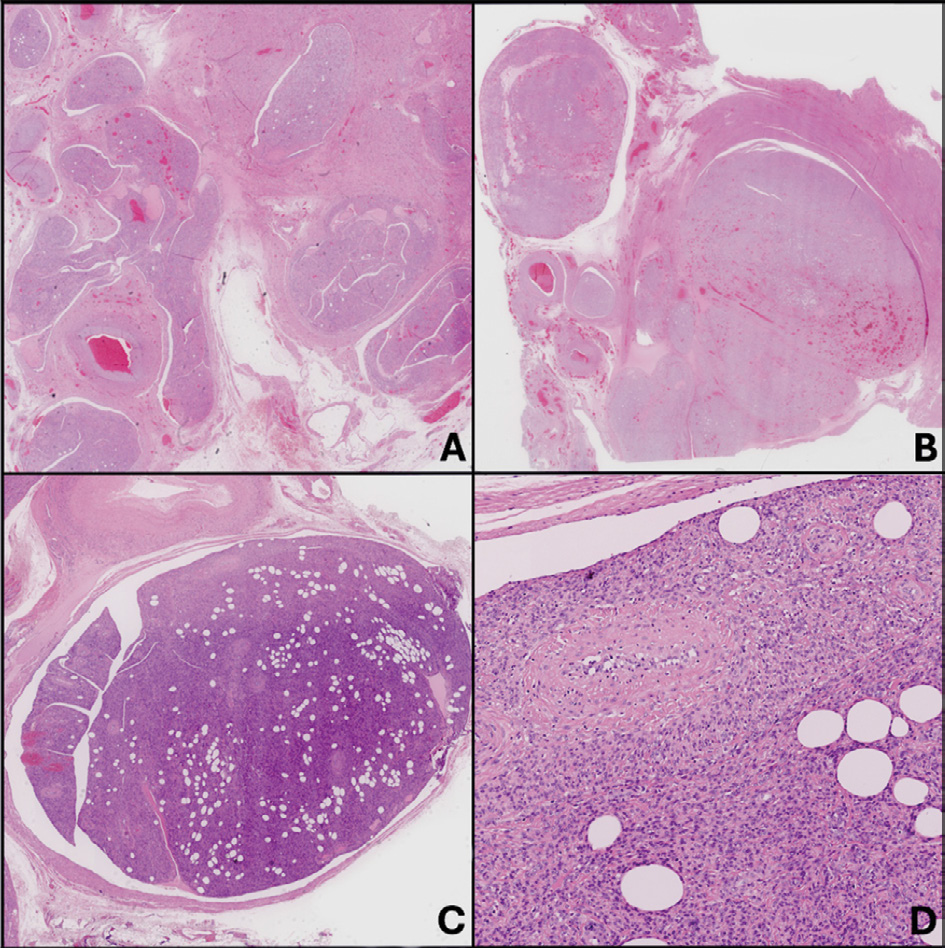
**A–D** images demonstrating a tumor growing predominantly within venous structures, showing a clefted intraluminal contour. The lesion contains thick-walled vessels, and the neoplastic cells exhibit smooth muscle and sometimes adipocytic differentiation. There is no increased mitotic activity, tumor necrosis, or cellular atypia, consistent with a diagnosis of intravenous leiomyomatosis, with extra-uterine extension (**B** and **C**).

Gross specimen (Figure 3) showed a uterus distorted by multiple nodules, extending into the left parametrium. Histopathology (Figure 4) revealed a smooth muscle tumor without atypia, necrosis, or significant mitotic activity. Intravascular proliferation involving uterine and pelvic vessels were also noted, confirming the diagnosis of IVL.

## Discussion

IVL often presents a challenging imaging diagnosis. MRI usually shows a tumor with a wormlike appearance and variable signal intensity on T1 and T2-weighted images, as signal intensity depends on the number of smooth muscle cells and fibrous tissue in the tumor. Following intravenous contrast administration, IVL may demonstrate either homogeneous or heterogeneous enhancement, which can further complicate interpretation, as its MRI appearance may mimic that of malignant uterine tumors.

In our case, MRI revealed a large heterogeneous myometrial mass with lobulated contours. Serpiginous extensions along parametrial and iliac veins matched the uterine mass, supporting intraluminal tumor. DWI/ADC showed no diffusion restriction, consistent with benign smooth muscle.

The main diagnostic differential in this context is low-grade endometrial stromal sarcoma (LG-ESS). LG-ESS may closely resemble IVL because it frequently demonstrates vascular permeation and extra-uterine spread. However, LG-ESS typically exhibits lower T2 signal intensity relative to myometrium, more infiltrative replacement of uterine tissue, and more evident diffusion restriction. In addition, LG-ESS usually shows early heterogeneous contrast enhancement, whereas IVL tends to demonstrate a more uniform enhancement pattern [4].

Other differential diagnoses include leiomyosarcoma, which typically demonstrates overt necrosis, hemorrhage with T1 hyperintensity, extra-uterine extension, and marked diffusion restriction [4]. Thrombosis is another differential diagnosis, characterized by the absence of enhancement and lack of continuity with a myometrial mass.

In this case, the pattern of venous extension and absence of diffusion restriction aligns with the classic imaging profile of IVL.

## Conclusion

IVL is a rare benign smooth muscle tumor that can mimic malignant uterine neoplasms on imaging. Recognition of serpiginous intravascular extensions continuous with a uterine mass and the absence of diffusion restriction is essential for raising suspicion for IVL. Awareness of these imaging findings aids in preoperative planning and ensures appropriate surgical management, while definitive diagnosis relies on histopathological confirmation.

All patient data have been completely anonymized throughout the manuscript and related files.
